# T Cell Production of GM-CSF Protects the Host during Experimental Tuberculosis

**DOI:** 10.1128/mBio.02087-17

**Published:** 2017-12-12

**Authors:** Richard T. Robinson

**Affiliations:** Department of Microbiology and Immunology, The Medical College of Wisconsin, Milwaukee, Wisconsin, USA

**Keywords:** CSF2, GM-CSF, GMCSF, immune, mycobacteria, tuberculosis

## Abstract

Although classically associated with myelopoiesis, granulocyte-macrophage colony-stimulating factor (GM-CSF) is increasingly recognized as being important for tuberculosis (TB) resistance. GM-CSF is expressed by nonhematopoietic and hematopoietic lineages following infection with *Mycobacterium tuberculosis* and is necessary to restrict *M. tuberculosis* growth in experimental models. Until the recent study by Rothchild et al. (mBio 8:e01514-17, 2017, https://doi.org/10.1128/mBio.01514-17), it was unknown whether GM-CSF-producing T cells contribute to TB resistance. Rothchild et al. identify which conventional and nonconventional T cell subsets produce GM-CSF during experimental TB, establish their protective nature using a variety of approaches, and provide a mechanistic basis for their ability to restrict *M. tuberculosis* growth. This commentary discusses the significance of these findings to basic and applied TB research. As translated to human disease, these findings suggest vaccine-mediated expansion of GM-CSF-producing T cells could be an effective prophylactic or therapeutic TB strategy.

## COMMENTARY

Colony-stimulating factors (CSFs) were first hypothesized to exist by Australian scientists following their classic observation that sera from a mouse strain with excessive myelopoiesis promote formation of “colonies” when added to *in vitro* bone marrow cultures (1). These colonies were sensitive to irradiation (2), comprised dense accumulations of progenitor cells, and gave rise to one or more phagocytic lineages (3). Members of the CSF cytokine family are positive regulators of myelopoiesis and include granulocyte CSF (G-CSF), macrophage CSF (M-CSF), and granulocyte-macrophage CSF (GM-CSF). It is now known that G-CSF and its receptor (G-CSFR) support neutrophil development during both “steady-state” and “emergency” granulopoiesis (4), whereas M-CSF and its receptor (M-CSFR) support the development and distribution of mononuclear phagocytes (5). Compared to G-CSF and M-CSF, the *in vivo* effects of GM-CSF and its receptor (GM-CSFR) on steady-state myelopoiesis are more limited. GM-CSF is a monomeric glycoprotein that is primarily secreted by epithelial cells during homeostasis; its roles during myelopoiesis are to promote development of alveolar macrophages (AMs) and nonlymphoid dendritic cells (DCs) (6).

In contrast to GM-CSF’s limited roles during steady-state myelopoiesis, it has many supportive roles during inflammation (6). Inflammation is a progressive immune response to substances that are nonself (e.g., infection) or self (e.g., autoimmunity) and is characterized by myeloid cell recruitment from the vasculature, accumulation in the affected tissue, and activation to clear the immunogenic substance. Although inflammation is critical for host protection from microbial infections, the physiological functions of all organs are negatively affected by inflammation. Myeloid responses during inflammation are sensitive to the activity of T cells (conventional and nonconventional) that are also present in the affected tissue. Whereas GM-CSF is primarily expressed by epithelial cells during homeostasis, both hematopoietic and nonhematopoietic lineages can express GM-CSF in an inflamed tissue (6). The local and systemic effects of GM-CSF follow its association with GM-CSFR, which comprises an α- and β-chain heterodimer on the cell surface; the GM-CSFR β-chain activates several signaling pathways (JAK-STAT, mitogen-activated protein kinase [MAPK], NF-κB, and phosphatidylinositol 3-kinase [PI3K]) that promote expression of multiple immune effector genes (7).

Inflammation has both protective and pathological roles in the mycobacterial disease tuberculosis (TB) (8). TB afflicts large portions of the globe and is caused by members of the *Mycobacterium tuberculosis* complex (*M. africanum*, *M. bovis*, *M. canettii*, *M. caprae*, *M. microti*, *M. mungi*, *M. pinnipedii*, *M. orygis*, and *M. tuberculosis*). *M. tuberculosis* is an intracellular pathogen that is transmitted via aerosolization of infected sputum. In most *M. tuberculosis*-infected individuals, *M. tuberculosis* does not cause clinical disease and persists in a slowly replicating or latent state. Maintaining *M. tuberculosis* in a latent state depends on inflammation, as the absence of select myeloid or T cell lineages—due to either an inherited or acquired immunodeficiency—predisposes humans to develop active or disseminated forms of TB. However, inflammation can also be damaging in the context of TB, as evidenced by the reduced survival and increased immunopathology observed in *M. tuberculosis*-infected PD1^−/−^ mice (9), interleukin-27^−/−^ (IL-27^−/−^) mice (10), and C57BL/6 mice following repeated *M. bovis* BCG vaccination (Koch’s phenomenon) (11). In *M. tuberculosis*-infected humans, the damaging effects of inflammation are exemplified by TB immune reconstitution inflammatory syndrome (TB-IRIS), which is due to a rapid expansion of immune cells in *M. tuberculosis*- and HIV-coinfected individuals following anti-HIV therapy (12).

GM-CSF expression is host protective in the context of TB infection. GM-CSF is expressed within human TB granulomas (13), is secreted by human macrophages and lung epithelial cells upon *M. tuberculosis* exposure (14–16), and reduces *M. tuberculosis* burden when added to infected human macrophage cultures (17). In the absence of GM-CSF, mice cannot restrict *M. tuberculosis* burden, are less capable of lymphocyte recruitment, and cannot form “normal” granulomas (18, 19). In addition to its pro-proliferative effects on AMs, GM-CSF increases the phagocytic capacity of AMs (20) and promotes the division of lung DCs that accumulate during TB (21). Since GM-CSF is produced by numerous hematopoietic and nonhematopoietic lineages during inflammation, what, if any, contribution T cell-derived GM-CSF has on TB outcome has been unknown. This is important to know for the following reason: if T cell-derived GM-CSF is protective, then vaccine-mediated expansion of GM-CSF-producing T cells could be an effective prophylactic or therapeutic TB strategy.

As they described (22), Rothchild et al. performed several adroit experiments to determine if GM-CSF from conventional and nonconventional T cells impacts TB outcome, as well as the signaling pathway through which this GM-CSF functions. Their results demonstrate GM-CSF protein levels mirror those of gamma interferon (IFN-γ) during TB progression; T cells are a major source of IFN-γ during TB, and T cell-derived IFN-γ promotes TB resistance (23). Similarly, T cell-derived GM-CSF also promotes TB resistance, as Rothchild et al. demonstrate using both adoptive transfer and radiation bone marrow chimera approaches. Among GM-CSF-producing T cells in mouse lungs, nonconventional T cells (i.e., invariant NKT [iNKT] cells and γδ T cells) vastly outnumber conventional T cells (CD4^+^ and CD8^+^ T cells) during the first 2 weeks postinfection; during later TB stages, conventional T cells are more represented among GM-CSF producers. GM-CSF-producing T cells are also present in the circulation of human TB patients (22, 24). To identify the mechanism through which GM-CSF limits *M. tuberculosis*, Rothchild et al. complement their animal studies with a cell culture model of TB (i.e., *M. tuberculosis*-infected macrophages). The latter studies demonstrate that GM-CSF alone can limit *M. tuberculosis* viability in a peroxisome proliferator-activated receptor gamma (PPARγ)-dependent manner and that GM-CSF’s effectiveness is higher in the presence of IFN-γ. PPARγ is a nuclear receptor that regulates gene transcription following macrophage recognition of *M. tuberculosis* mannose-capped lipoarabinomannan (MAN-LAM) (25). Collectively, the results from the study by Rothchild et al. establish the kinetics of T cell GM-CSF production during experimental TB, quantify conventional and nonconventional T cells’ contribution to GM-CSF levels at each disease stage, establish the protective nature of GM-CSF-producing T cells, and provide a mechanistic basis for their protective capacity.

One reason the study by Rothchild et al. (22) is significant is there is now a basis for determining if expanding the number of GM-CSF-producing T cells (which have been dubbed “T_H_5” cells given their dependence on STAT5 activity [26]) is an effective vaccine strategy. T and B cells are the basis of vaccine-elicited immune memory; therefore, unlike GM-CSF-producing innate lineages, GM-CSF-producing T cells could theoretically be induced via vaccination to facilitate TB resistance. Selective expansion of GM-CSF-producing T cells will depend on identification of factors that induce differentiation of GM-CSF-producing T cells. These factors likely include novel transcription factors (e.g., DEC1 or Bhlhe40) that have been identified in other disease models (6). Given that the antimicrobial effects of GM-CSF and IFN-γ are additive (22), an important future direction will be to determine if T cells producing both GM-CSF and IFN-γ are more protective than their single-positive counterparts and to identify which chemokines promote their localization into tubercular tissue. Also, in the same way that excessive T_H_17 cell numbers can damage *M. tuberculosis*-infected lungs (11), it will be important to determine if an excessive T cell GM-CSF response tips the balance between protective versus damaging inflammation.

Finally, the study by Rothchild et al. (22) is also important because it has implications for using GM-CSF as an adjunct TB therapy. There is an urgent need for adjunct therapies that either shorten TB treatment or improve TB outcome (27). Since the discovery of GM-CSF has Australian roots (3), it is appropriate that Australian scientists were also the first to test GM-CSF’s therapeutic capacity in *M. tuberculosis*-infected animals (28), with several groups continuing related treatment studies (29–32). Rothchild et al. (22) demonstrated that GM-CSF production by either radioresistant or radiosensitive cells promotes TB resistance, which supports ongoing efforts to increase GM-CSF levels in TB patients—either by administering recombinant GM-CSF (i.e., sargramostim) or by enhancing endogenous GM-CSF expression (e.g., via gene therapy)—to positively affect TB outcome. Adult TB patients treated with adjunct subcutaneous GM-CSF trend toward faster sputum clearance with minimal, transient side effects (33). Whether pediatric TB patients can be effectively treated with adjunct GM-CSF has not yet been reported, but adjunctive GM-CSF was recently used to successfully treat invasive fungal infection in a child (34). In addition to supporting the use of GM-CSF therapy for TB, the study by Rothchild et al. (22) provides a basis for exploring whether GM-CSF–IFN-γ coadministration is an effective adjunct TB therapy. Recombinant IFN-γ treatment is used to prevent infection in patients with chronic granulomatous disease (CGD), albeit with negative side effects (flu-like symptoms); its efficacy as an adjunct TB therapy has also been tested in small clinical trials, with positive results (27). Given the observation by Rothchild et al. (22) that GM-CSF and IFN-γ additively affect *M. tuberculosis* growth, it is possible that GM-CSF–IFN-γ coadministration would lower the IFN-γ minimal effective dose (i.e., maintain IFN-γ efficacy as an adjunct TB therapy) while also attenuating its negative side effects. Although years may pass before this possibility is formally tested in a clinical trial, and translating animal model data to human TB therapy has recognized limitations, the results from the study by Rothchild et al. (22) nonetheless provide a scientific basis for exploring GM-CSF–IFN-γ as a cytokine combination therapy for TB.
